# Review

**DOI:** 10.1186/1743-1050-5-3

**Published:** 2008-06-10

**Authors:** Eric Scott Sills

**Affiliations:** 1 The Sims Institute, Rosemount Hall, Dundrum Road, Dublin 14, Ireland

Perhaps the only remaining non-controversial topic in IVF is the undisputable impact of embryo transfer on reproductive outcome. Nevertheless, this key convergence of art and science resists easy and objective critique.

In *Embryo Transfer *(Anshan Publishers, UK; 2008), Allahbadia meets this challenge by bringing together a new comprehensive monograph that lays a calliper across the full range of the embryo transfer phenomenon.

The book thoughtfully covers all aspects of ET with the benefit of an international cast of experts. The book's 54 chapters have minimal content overlap and are organised into 13 logical sections. The text is nicely written and well edited. Each chapter opens with a concise summary – a feature busy clinicians will no doubt find particularly helpful.

Extensive data are presented comparing both rigid and soft ET catheters, and readers will also find tips on catheter loading, cervical preparation, and embryo placement at transfer.

Transmyometrial ET is also included under 'difficult transfer' and receives an authoritative discussion. The chapter on the complexities of luteal phase P_4 _support is ambitious and tempting; this subject alone could easily constitute its own companion monograph as a sequel. And for those doubting the relevance of ultrasound-guided ET, balanced but compelling evidence shows this is the future of IVF.

With a tone too approachable for staid textbook status, the book nevertheless is a scholarly work and exhaustively referenced. Its size serves it well as a ready reference. The cover is handsomely designed with a whimsical depiction of a three-embryo transfer as a nod to our shared history.

If the art of successful ET can be likened to a symphonic finale, this important opus provides some elegant lessons from talented virtuosi.

